# Short- and Long-term Outcomes of an Acellular Dermal Substitute versus Standard of Care in Burns and Reconstructions: A Phase I/II Intrapatient Randomized Controlled Trial

**DOI:** 10.1097/ASW.0000000000000040

**Published:** 2023-09-14

**Authors:** Kim L. M. Gardien, Anouk Pijpe, Katrien M. Brouwer, Matthea Stoop, Simarjeet K. Singh, Floyd W. Timmermans, Marcel Vlig, Paul P. M. van Zuijlen, Esther Middelkoop

**Affiliations:** Kim L. M. Gardien, MD, is Burn Physician and Anouk Pijpe, PhD, is Epidemiologist and Research Coordinator, Association of Dutch Burn Centres, Beverwijk, the Netherlands; Burn Center, Red Cross Hospital, Beverwijk; Department of Plastic, Reconstructive and Hand Surgery, Amsterdam UMC, Vrije Universiteit Amsterdam; and Amsterdam Movement Sciences (AMS) Institute, Amsterdam UMC. Katrien M. Brouwer, PhD, is Senior Researcher, Association of Dutch Burn Centres. Matthea Stoop, RN, is Research Nurse, Association of Dutch Burn Centres; Burn Center, Red Cross Hospital, Beverwijk; Department of Plastic, Reconstructive and Hand Surgery, Amsterdam UMC, Vrije Universiteit Amsterdam. Simarjeet K. Singh is Research Student, Burn Center, Red Cross Hospital, Beverwijk. Floyd W. Timmermans, MD, PhD, is Clinical Researcher, Department of Plastic, Reconstructive and Hand Surgery, Amsterdam UMC, Vrije Universiteit Amsterdam; and AMS Institute, Amsterdam UMC. Marcel Vlig, BAS, is Senior Technician, Association of Dutch Burn Centres. Paul P. M. van Zuijlen, MD, PhD, is Plastic Surgeon and Professor of Burn Care, Burn Center, Red Cross Hospital, Beverwijk; Department of Plastic, Reconstructive and Hand Surgery, Amsterdam UMC, Vrije Universiteit Amsterdam; AMS Institute, Amsterdam UMC; and Department of Plastic, Reconstructive and Hand Surgery, Red Cross Hospital, Beverwijk. Esther Middelkoop, PhD, is Director of Research and Professor of Wound Healing and Skin Regeneration, Association of Dutch Burn Centres, Beverwijk, the Netherlands; Burn Center, Red Cross Hospital, Beverwijk; Department of Plastic, Reconstructive and Hand Surgery, Amsterdam UMC, Vrije Universiteit Amsterdam; and AMS Institute, Amsterdam UMC.

**Keywords:** artificial skin, burns, dermal substitute, reconstruction, scar quality, wound healing

## Abstract

**OBJECTIVE:**

Dermal substitutes promote dermal regeneration and improve scar quality, but knowledge gaps remain regarding their efficacy and indications for use. The authors investigated the safety and short- and long-term efficacy of an acellular dermal substitute in patients with full-thickness wounds.

**METHODS:**

This intrapatient randomized controlled, open-label, phase I (safety) and phase II (efficacy) study compared treatment with Novomaix (Matricel GmbH), a dermal collagen/elastin-based scaffold, with split-thickness skin graft (STSG) only. The primary safety outcome was graft take at 5 to 7 days postsurgery. Postsurgical scar quality was assessed by measuring elasticity, color, and scores on the Patient and Observer Scar Assessment Scale at 3 months, 12 months, and 6 years.

**RESULTS:**

Twenty-five patients were included, of which 24 received treatment allocation. Graft take and wound healing were statistically significantly lower/delayed in the dermal matrix group compared with STSG alone (*P* < .004). Serious adverse events were delayed epithelialization in four dermal matrix and three STSG study areas. At 12 months postsurgery, skin extension (*P* = .034) and elasticity (*P* = .036) were better for the dermal matrix group compared with the group receiving STSG alone. Other scar quality parameters at 12 months and 6 years did not differ between treatment arms.

**CONCLUSIONS:**

The dermal substitute was a safe treatment modality for full-thickness wounds. Compared with STSG alone, time to wound healing was slightly increased. Nevertheless, scar quality at 12 months seemed somewhat improved in the wounds treated with the dermal substitute, indicative of enhanced scar maturation. In the long term, final scar quality was similar for both treatment modalities.

## INTRODUCTION

Dermal substitutes have emerged as valuable therapeutic options in the treatment of full-thickness wounds. Generally, the standard of care for these wounds consists of full-thickness skin graft (FTG) coverage where possible or split-thickness skin grafts (STSG) when needed. Dermal substitutes enable dermal replacement without requiring the harvesting of full-thickness skin. The poor scar quality and donor site morbidity seen after autologous STSGs, the limited availability of and morbidity seen in FTG, and the understanding of the importance of dermal regeneration for final outcomes have incentivized the further development of skin-replacement therapies. In practice, alternatives are needed that enable immediate wound coverage and enhance wound closure, while also reducing the need for FTG or STSG harvesting. Specific considerations validate the use of these substitutes, such as the location and depth of burns and donor site availability, with the aim of improving scar quality.

Despite the availability of many different dermal substitutes, few high-quality clinical studies including long-term outcomes in burn and reconstructive patients are available.^1–5^ In a systematic review, Widjaja et al^6^ noted that only a few clinical studies had examined the efficacy of dermal substitutes, and these all studied different products. They concluded that more randomized controlled trials were needed to strengthen the evidence of efficacy and support the rationale for use. Further, dermal substitutes are used in a wide variety of clinical indications, such as burns and reconstructive surgery; oral, abdominal wall, and breast reconstructive surgery; and chronic wound management.^7–9^ Which dermal substitute is best suited for which indication is an ongoing subject of research.^9^

Research has already examined the efficacy of dermal substitutes in improving scar quality parameters in acute and reconstructive wounds.^1,10^ Van Zuijlen et al^10^ found that with dermal substitute use, graft take, skin color, and various scar elasticity parameters were improved after 3 months. However, graft take for burns was slightly lower when using a dermal substitute in a one-step procedure, most likely due to impaired ingrowth of autologous cells.^10^ Efforts to improve graft take have included pore size adaptations to improve cell migration, simultaneous application of topical negative pressure,^11^ and cross-linking to increase the stability of the product.^12–15^

To overcome some of the known limitations of dermal substitutes, a novel dermal substitute (Novomaix; Matricel GmbH) was developed that consists of porcine collagen and elastin with a unidirectional pore structure. This pore structure may facilitate cellular ingrowth. In a porcine wound model, Boekema et al^16^ reported this dermal substitute enabled one-stage grafting with STSG, resulting in good wound healing and a very mild foreign body reaction. They concluded that the substitute was a promising candidate for further clinical evaluation.^16^

Therefore, the authors’ aim in the present study was to investigate the safety and short- and long-term efficacy of this dermal substitute in patients with acute burns and reconstructive wounds. This study is the first clinical application of dermal matrix in acute and reconstructive wounds and is designed as phase I and phase II trials.

## METHODS

### Study Design and Patient Selection

This study was a prospective, open-label intrapatient randomized controlled trial performed at the Red Cross Hospital in Beverwijk, the Netherlands. The objectives of this study were to evaluate the short- and long-term first-in-man safety (phase I) and efficacy (phase II) of an acellular dermal substitute and compare the outcomes with the standard of care, which was 1:1.5 ratio meshed STSG in full-thickness skin defects. The authors used an intrapatient comparison model because wound-healing characteristics and scar formation are generally highly patient specific. Two comparable wound areas were chosen, one wound area was covered with dermal matrix on top of which a 1:1.5 ratio meshed STSG was placed, and the other wound area was covered with the conventional 1:1.5 ratio meshed STSG only. The allocation of treatment was randomized.

The trials were performed in accordance with the Declaration of Helsinki, Guidelines for Good Clinical Practice, and the CONSORT (Consolidated Standards of Reporting Trials) statement for reporting within-person randomized trials.^17^ The study protocols of the phase I and phase II trials were approved by the Medical Ethics Committee of the district Noord-Holland in the Netherlands (NL42113.094.12 and NL50542.094.14) and the institutional review board of the Red Cross Hospital and registered at Clinical Trials (NCT02164760 and NCT02373566). The long-term follow-up study was approved by the Medical Ethics Review Committee of VU University Medical Center (2020.426) and the institutional review board of the Red Cross Hospital.

**Figure 1.**
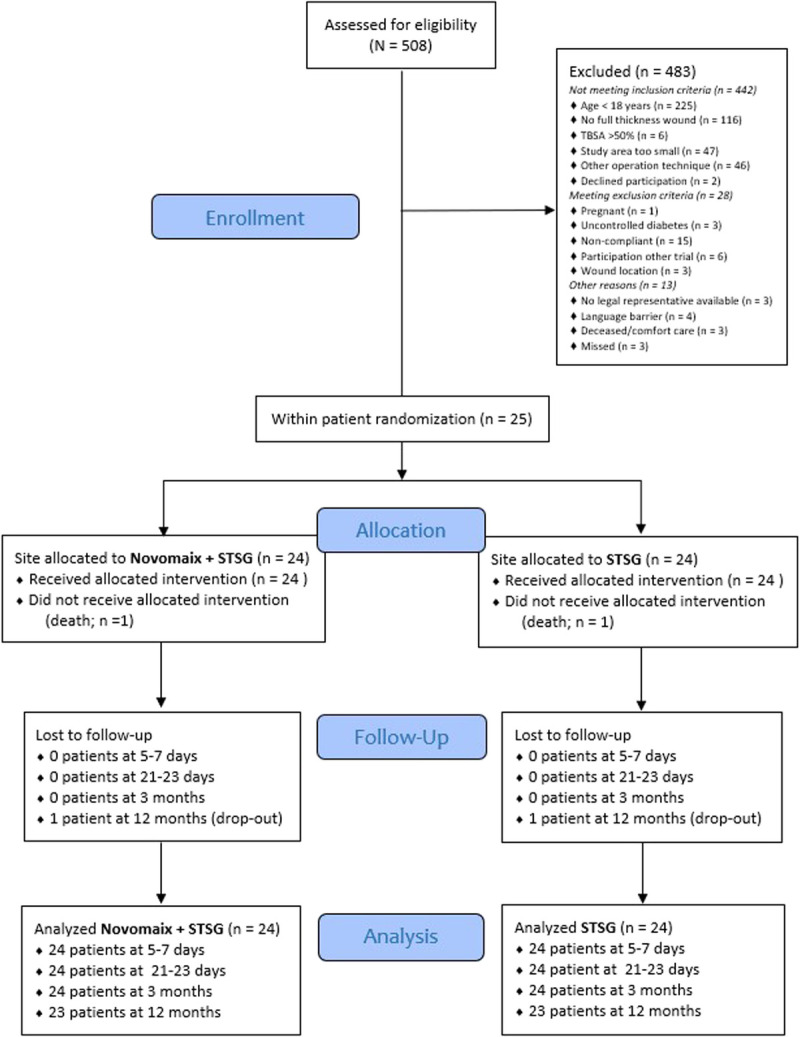
CONSORT FLOW DIAGRAM OF THE PHASE I AND PHASE II TRIALS (COMBINED) Abbreviation: STSG, split-thickness skin graft.

Patients with acute full-thickness burn wounds that required skin grafting or scars requiring reconstructive surgery who were seen between June 2013 and April 2014 were included in the phase I trial, and those seen between March 2015 and August 2016 were included in the phase II trial. Because the phase I trial analysis confirmed that the dermal substitute combined with meshed STSG is a safe wound treatment modality, the authors continued with the phase II efficacy trial. For the purpose of maximum power, the data from the phase I and II studies were pooled, and the pooled results are presented. The CONSORT flow diagram is shown in Figure 1.

All admitted patients were checked for eligibility according to the inclusion and exclusion criteria (Table 1). For the long-term follow-up study conducted between October 2020 and December 2021, all patients included in the phase I and phase II trial were eligible, when still alive and traceable. Patients were contacted through a study information letter, followed by a phone call. Nonresponding patients were sent a reminder.

**Table 1.** INCLUSION AND EXCLUSION CRITERIA

Inclusion Criteria	Exclusion Criteria
- Informed consent by the patient and/or legal representatives - Age ≥18 y with full-thickness skin defects that require skin grafting AND - ≤50% Total body surface area burned with full-thickness skin defects at time of intervention AND - Full-thickness skin defects configured in such a way that two comparable and measurable areas can be grafted, both a minimum of 3 × 3 cm	- Patients with infected wounds - Patients who are pregnant or breastfeeding - Patients with known concomitant medical conditions that may interfere with normal wound healing (eg, immune deficiency, HIV, uncontrolled diabetes, cancer, etc) - Known allergy to porcine collagen or elastin - Patients who are expected (according to the responsible medical doctor) to be nonadherent to the study protocol - Previous enrollment of the patient into the current study - Full-thickness skin wound located in face and/or genitals

In all three trials, all participants provided written informed consent before participation.

### Study Materials

The dermal substitute Novomaix regeneration matrix is an open, porous sponge-based acellular biodegradable scaffold prepared from fibrillar type I and type III collagen and elastin fibers isolated from highly purified porcine tissue. The pore structure is prepared using a directional freezing technology that creates longitudinal pores as guiding structures for dermal regeneration.^18,19^ In this study, the authors used sheets of dermal matrix that were 5 × 5 cm with a thickness of 1 mm.

### Randomization and Blinding

Prior to randomization, the surgeon selected two similar wound areas. Treatment allocation of the experimental and control sites was concealed in an envelope, which remained sealed up until the stage of wound bed coverage. The operated areas were thus randomly treated with either the dermal matrix treatment (dermal matrix plus STSG) or the control treatment (STSG alone). Surgeons were not blinded to the allocated treatment. The patients and researcher were blinded to treatment allocation, although at early time points dermal matrix may have remained visible. To reduce bias, follow-up measurements were not performed by the surgeon who performed the surgery, but by trained researchers or nurses.

### Study Procedure

The wound bed was prepared according to routine procedures (eg, tangential excision [Humby knife] and/or hydrojet techniques [Versajet; Smith & Nephew]) for acute wounds and sharp excision for reconstructive wounds. The STSG was harvested using an electric Zimmer dermatome (Zimmer Inc) and expanded to a 1:1.5 ratio by mesh technique. The dermal matrix was placed onto the wound bed and hydrated with sterile 0.9% sodium chloride solution. The surgeon applied the dermal matrix and trimmed the material to align with the wound edges. Air inclusions between the wound bed and the product were removed because they could impair diffusion of nutrients and cell migration and prevent proper adhesion. The surgeon fixed the STSG on both the dermal matrix and the control treatment area using staples. Postoperative wound management was identical for both wound areas using a nonadherent membranous dressing. Follow-up moments in the phase I and phase II trials were at 5 to 7 days, 21 days, 3 months, and 12 months after surgery. In the long-term follow-up study, the mean time postsurgery was 71 (range, 49–86) months.

### Safety Outcomes

#### Graft take, wound healing, and serious adverse events (SAEs)

The primary outcome in the phase I trial was graft take of the dermal matrix versus the control area at 5 to 7 days postsurgery. Assessment of wound epithelialization after the initial phase of graft take was used as a marker for undisturbed wound healing. Other wound-healing parameters included percent (%) epithelialization at 21 to 23 days after surgery and time required (in days) to reach complete wound closure. Experienced clinicians monitored and assessed graft take and wound-healing parameters. For the duration of the phase I and phase II trials, the occurrence of SAEs was also monitored.

#### Microbiology

During the clinical admission period, wound swabs were taken routinely twice weekly. Wound swabs were also taken preoperatively on the day of operation. The randomization procedure took place after wound bed preparation. Therefore, the wound swabs taken preoperatively represent the total area of interest.

### Efficacy Outcomes

The same standardized and validated set of scar quality outcome measurement instruments was used in the phase I, phase II, and long-term follow-up study periods. Scar quality was measured at 3, 12, and a mean of 71 months postsurgery.

#### Elasticity measurement

Scar elasticity was measured with a validated instrument, the Cutometer (Courage & Khazaka GmbH).^20^ The results are shown as the ratio of scar elasticity between scar tissue and nonaffected skin of an anatomically comparable area for either the dermal matrix or standard-treated area. Values closer to 1 indicate more comparable elasticity to that of normal skin. The primary outcome measure in the phase II efficacy trial was the elasticity (final deformation Uf measured by Cutometer) at 3 months postsurgery.

#### Scar assessment

The Patient and Observer Scar Assessment Scale (POSAS) 2.0 is a reliable and validated scale that consists of two numeric scales: the Patient Scar Assessment Scale and the Observer Scar Assessment Scale.^21–24^ The six parameters of the patient scale are pain, itching, color, stiffness, thickness, and surface irregularity. The six parameters of the observer scale are vascularization, pigmentation, thickness, relief, pliability, and surface area. Responses were gathered into a score ranging from 1 to 10 for each parameter for a total summed score between 6 (representing “normal skin”) and 60 (“worst imaginable scar”).

#### Color measurement

The DermaSpectrometer (DSM-II) (Cortex Technology) is a validated instrument to measure scar vascularization (erythema) and pigmentation (melanin) by a narrowband simple reflectance meter.^25,26^ The scar erythema index and melanin index were assessed and calculated as the absolute difference between scar tissue and the nonaffected skin of the patient.

#### Scar preference

The patients’ scar preference was determined by asking the patient which scar area they preferred (study area A, study area B, or no preference) at 3 months, 12 months, and 6 years.

### Statistical Analysis

Statistical analyses were performed using SPSS version 25 (IBM Corp). No a priori sample size calculation was possible for the phase I study. For the phase II study, the estimated minimum required sample size was 20 patients, including the phase I group. Continuous variables are presented as mean and SD in case of a normal distribution and as median and interquartile range (25th, 75th percentile) for variables with a nonnormal distribution. Categorical variables are presented as frequencies and percentages. Differences between the dermal matrix and control sites were analyzed by a paired-sample *t* test or, in the case of nonparametric data, the paired Wilcoxon signed-rank test. *P* < .05 was considered statistically significant.

## RESULTS

### Patient Population

A total of 25 patients were included, of which 13 were part of the phase I study. The CONSORT flowchart for intrapatient controlled trials is provided as Figure 1. The patient, wound, and treatment characteristics are presented in Table 2. Within the pooled group that received treatment allocation (n = 24; one patient died prior to randomization), 13 were male and 11 were female. Indications for surgery were reconstructive wounds in 5 participants (21%) and acute burns in 19 participants (79%) with a median total burned surface area of 8% (range, 2%–67%). No significant differences were noted between patient characteristics in the phase I versus phase II group.

**Table 2.** PATIENT AND TREATMENT CHARACTERISTICS

Characteristic	Value
No. of included patients	25
No. of randomized patients	24^a^
Male/female, n	13/11
Age at surgery, y, median (range)	53.5 (19-87)
Type of wound, n (%)	
Reconstructive wound	5 (21)
Acute burn wound	19 (79)
- TBSA full thickness, %, median (range)	5.0 (1-52)
- TBSA total, %, median (range)	8.0 (2-67)
Etiology,^b^ n (%)	
Flame	12 (63)
Fat	3 (16)
Bioethanol	2 (11)
Contact	1 (5)
Scald	1 (5)
Postburn day of operation,^b^ median (range)	11.0 (7-59)
Study location, n (%)	
Arm	7 (29)
Trunk	6 (25)
Leg	6 (25)
Hand	2 (8)
Neck	2 (8)
Foot	1 (4)
Wound bed preparation, n (%)	
CTE	8 (33)
Versajet	3 (13)
Versajet + CTE	7 (29)
NexoBrid + Versajet	1 (4)
Scar release	5 (21)
First postoperative wound dressing/treatment, n (%)	
UrgoTul + AgNO_3_-PEG 0.5%	2 (8)
UrgoTul + PEG Betadine	4 (17)
UrgoTul + Furacine	10 (42)
UrgoTul SSD	7 (29)
Topical negative pressure + Cuticell	1 (4%)

Abbreviation: CTE, conventional tangential excision; PEG, polyethylene glycol; SSD, silver sulfadiazine; TBSA, total body surface area.

^a^One patient died between inclusion and randomization.

^b^Acute burn wounds only (n = 19).

In the years after the phase I and II trials, three patients died, and one emigrated, so 20 patients were eligible for the long-term follow-up study. Among those invited, one patient was not interested in participation and four patients did not respond to the invitation, resulting in the inclusion of 15 patients (76% response rate). These patients did not differ from the original study population in any of the patient and treatment characteristics (comparative analysis not shown).

### Safety Outcomes

#### Graft take and wound healing

**Figure 2.**
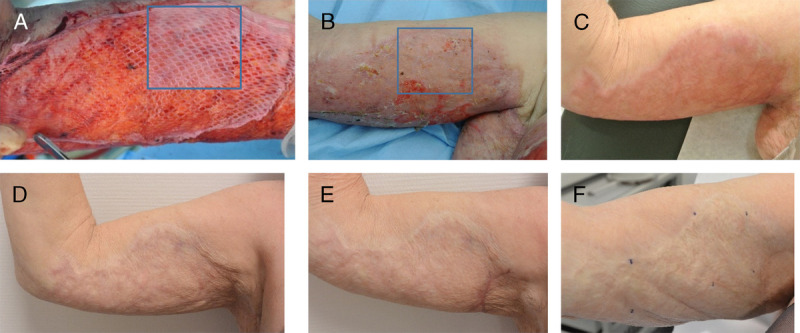
TREATMENT OF A STUDY PATIENT Acute burn wound (flame burn) at 12 days postburn. A, Perioperative placement of the dermal substitute (indicated by square) onto the wound bed with an STSG on top of the dermal substitute. B, Wound healing of both study areas at 12 days postoperative. C, Scar quality at 3 months postoperative. D, Scar quality at 1 year postoperative. (E) Scar quality at 1.5 years postoperative. F, Scar quality after 6.5 years. Abbreviation: STSG, split-thickness skin graft. Note: The patient provided permission for these images to be published.

The graft take of the dermal matrix wound and standard treated wound at days 5 to 7 was 80% and 95%, respectively (*P* = .003; Table 3). The epithelialization rate of the dermal matrix wound area at this time point was 70% compared with 89% for the control wound treatment (*P* < .001). At 21 to 23 days after surgery, the re-epithelialization rate was 80% for the dermal matrix treatment compared with 96% for the standard treatment (*P* < .001). The time to complete wound healing (≥95%) was 28 days for the dermal matrix treatment and 16 days for the standard treatment (*P* = .004). Subgroup analysis of acute and reconstructive wounds offered similar results, albeit that for the five reconstructive wounds, graft take and time to wound healing were not statistically different between wounds treated with dermal matrix versus standard of care (not shown). The course of wound healing and outcome of scar formation are illustrated in Figure 2.

**Table 3.** GRAFT TAKE AND WOUND EPITHELIALIZATION (n = 24)

Variable	Dermal Matrix, Median (Range)	Standard Care, Median (Range)	*P* **^a^**
Graft take 5–7 d postsurgery, %	80 (30–100)	95 (63–100)	.003
Epithelialization 5–7 d postsurgery, %	70 (5.0–92.5)	89 (53–98)	<.001
Epithelialization 21–23 d postsurgery, %	80 (10–100)	96 (45–100)	<.001
Complete wound healing postsurgery, d	28 (4–94)	16 (4–48)	.004

^a^Paired-samples Wilcoxon signed-rank test.

#### SAEs

Nine SAEs occurred during the phase I and II trials and were reported to the Medical Ethics Committee of the district Noord-Holland in the Netherlands. All SAEs occurred in patients with acute burn wounds. One SAE concerned a patient who died before application of the investigational product. Of the eight remaining SAEs, five were related to the study areas and concerned slow wound healing, for which reoperation was deemed beneficial. Of these five, two patients experienced wound colonization/infection in which the dermal matrix area was reoperated, one patient was reoperated in the standard treatment area because of shearing forces in the wound area, and two patients were reoperated in both areas because of slow wound healing. The other three SAEs concerned a reoperation to stimulate wound healing in areas other than the study areas.

#### Microbiology

Wound cultures were defined as positive if one or more of the following species were found: *Staphylococcus aureus, Proteus mirabilis, Escherichia coli, Enterobacter cloacae complex, Pseudomonas aeruginosa, Acinetobacter lwoffii, Acinetobacter baumannii,* or *Achromobacter xylosoxidans*. Preoperatively, positive cultures were detected in 7 of 24 patients. Postoperatively, 13 patients had positive bacterial cultures in both study wounds, and 2 patients had positive bacterial cultures only in the dermal matrix-treated area. All patients received either preventive or therapeutic topical antibacterial treatment. Local wound colonizations or infections were treated using UrgoTul (Urgo Medical) in combination with an antimicrobial component as the standard postoperative wound dressing (Table 2). Depending on the wound culture results and clinical signs, wounds were treated topically using nitrofural, fusidic acid, mupirocin, colistimethate, silver nitrate solution, or povidone-iodine cream. Topical treatment generally was continued until wound closure. Mean duration was 28 days (including the initial postsurgical wound dressing period with UrgoTul).

### Efficacy Outcomes

#### Elasticity measurement (Cutometer)

A statistically significantly higher elasticity ratio (indicating a better comparability to normal skin) was found in the dermal matrix-treated area in comparison with the standard treated area for the parameters Uf (final skin deformation, *P* = .034)) and Ue (skin extensibility, *P* = .036) as measured by the Cutometer at 12 months’ follow-up. No statistically significant differences were observed at 3 months or 6 years (Table 4) or for the other Cutometer parameters (data not shown).

**Table 4.** SCAR ELASTICITY AS ASSESSED BY THE CUTOMETER

Cutometer Parameter**^a^**	Dermal Matrix, Mean (SD)	Standard Care, Mean (SD)	*P* **^b^**
**Uf (final deformation)**			
3 mo (n = 23)^c^	0.48 (0.18)	0.45 (0.16)	.441
12 mo (n = 22)^c,d^	0.69 (0.26)	0.59 (0.19)	.034
71 mo (n = 14)^c^	0.60 (0.24)	0.63 (0.21)	.300
**Ue (skin extensibility)**			
3 mo (n = 23)	0.42 (0.17)	0.40 (0.17)	.659
12 mo (n = 22)	0.66 (0.29)	0.54 (0.19)	.036
71 mo (n = 14)	0.57 (0.23)	0.59 (0.20)	.536

^a^Data are presented as mean ratio of the respective scar elasticity versus normal uninjured skin.

^b^All parameters are tested by paired-samples *t* test.

^c^Cutometer assessment was not possible in one patient because of extreme stiffness of the scars.

^d^One patient was lost to follow-up at 12 months’ evaluation.

#### Scar assessment

**Figure 3.**
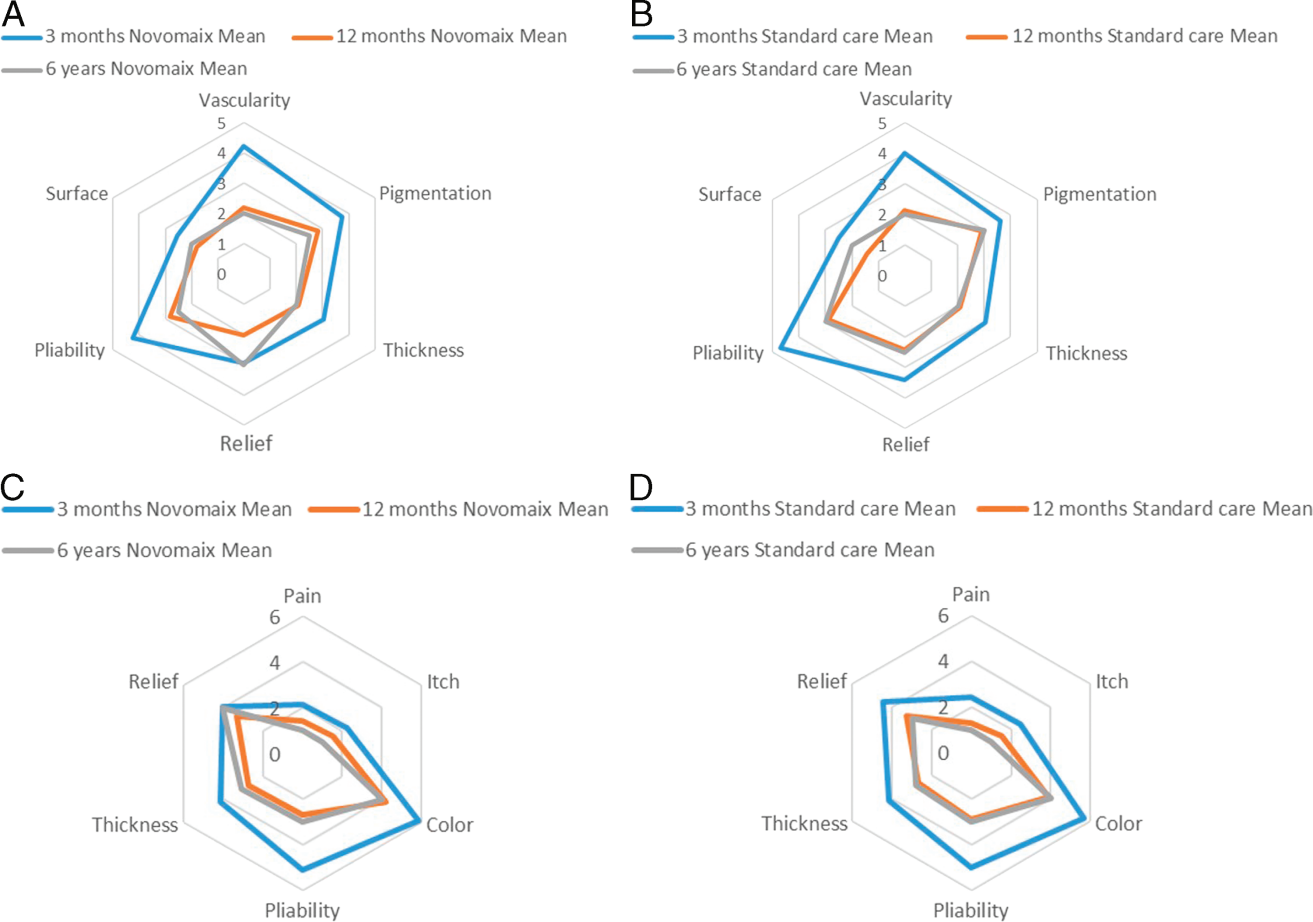
POSAS OBSERVERS SCORES FOR DERMAL MATRIX AND STANDARD OF CARE TREATMENT OVER TIME A, POSAS observer scores for dermal matrix-treated wounds at 3 months, 12 months, and 6 years posttransplantation. B, POSAS observer scores for standard of care-treated wounds at 3 months, 12 months, and 6 years posttransplantation. C, POSAS patient scores for dermal matrix-treated wounds at 3 months, 12 months, and 6 years posttransplantation. D, POSAS patient scores for standard of care-treated wounds at 3 months, 12 months, and 6 years posttransplantation. Abbreviation: POSAS, Patient and Observer Scar Assessment Scale. Note: Scores closer to 1 indicate more similarity to normal, uninjured skin.

For all three assessment time points, the dermal matrix and standard treatment did not differ in the mean observer POSAS scores; the mean score was 3.5 at 3 months, 2.3 at 12 months, and 2.8 at 6 years for both treatment areas (Table 5 and Figure 3). A similar pattern was observed for the mean patient POSAS scores. A trend was observed for the items “pliability” and “relief” in the observer scale and “pain” in the patient scale, in favor of the dermal matrix-treated area (*P* = .076, *P* = .080, and *P* = .059 respectively); at 12 months, the item surface in the observer scale was statistically significantly rated more favorable for standard care over dermal matrix (1.43 vs 1.78, respectively, *P* = .048; Supplementary Table, http://links.lww.com/NSW/A161). No other statistically significant differences were observed for any of the items in the observer or patient scales for all three assessment time points (Supplementary Table).

**Table 5.** SCAR QUALITY AS ASSESSED BY THE POSAS

POSAS	n	Dermal Matrix, Mean (SD)	Standard Care, Mean (SD)	*P*
**Observer, mo postsurgery**				
3	24	3.45 (1.02)	3.55 (0.81)	.556^a^
12	23	2.29 (0.83)	2.30 (0.69)	.952^a^
71	15	2.77 (1.18)	2.73 (1.13)	.769^a^
**Patient, mo postsurgery**				
3	24	3.98 (1.23)	4.05 (1.14)	.686^a^
12	23	2.88 (1.57)	2.74 (1.27)	.975
71	15	2.86 (1.55)	2.63 (1.10)	.333

Abbreviation: POSAS, Patient and Observer Scar Assessment Scale.

^a^Paired-samples *t* test; for the other parameters, the Wilcoxon matched-pair signed-rank test was used.

#### Color measurement (DSM-II)

No significant differences between the dermal matrix and the standard treatment were found for the erythema and melanin index measured with the DSM-II at the 3-month, 12-month, or 6-year follow-ups (Table 6).

**Table 6.** SCAR COLOR AS ASSESSED BY THE DSM-II DERMASPECTROMETER

Variable	n	Dermal Matrix, Mean (SD)	Standard Care, Mean (SD)	*P* **^a^**
**Erythema, mo postsurgery**				
3	24	6.56 (3.11)	6.57 (3.70)	.982
12	23	4.05 (3.15)	3.40 (1.92)	.271
71	15	2.82 (2.86)	2.17 (2.12)	.112
**Melanin, mo postsurgery**				
3	24	13.17 (6.13)	15.38 (7.65)	.218
12	23	9.51 (7.47)	10.03 (7.43)	.524
71	15	10.01 (9.69)	8.24 (7.75)	.140

Note: Data are presented as the mean difference of the absolute values of the respective scar versus normal uninjured skin.

^a^Paired-samples *t* test for difference at 3 and 12 months; Wilcoxon signed-rank test for difference at 71 months.

#### Scar preference

At 3 months, almost half (46%) of the patients had no preference for a scar area, 25% preferred the dermal matrix area, and 29% preferred the standard treatment area. At 12 months, 39% of the patients preferred the dermal matrix area, whereas the rest of the patients preferred the standard treated area (30%) or had no preference (30%). In the long-term study, the majority of patients had no preference (47%) or preferred the dermal matrix area (40%); only two patients (13%) preferred the standard treated area.

## DISCUSSION

This safety and efficacy study presents the first results of the use of a novel dermal substitute in patients with burn and reconstructive wounds. The results of this study show that, similar to other known dermal substitutes, early wound healing was delayed in comparison with STSG alone.^10,27,28^ This is likely due to the interpositioning of a dermal substitute, which delays the initial take of the graft and epithelialization.

Slow wound healing in the study areas was a reason to reoperate in five patients; in two cases, this concerned only the dermal matrix area, and in one case, only the standard treatment area was reoperated. It should be noted that reoperations are relatively frequent in patients with large burn wounds. This is illustrated by the fact that reoperations took place in three other patients because of slow wound healing, but these areas were not related to the study areas. Bacterial colonization in the dermal matrix-treated areas was possibly related to the extended wound-healing time. This was noted in 2 of 24 cases; in all other cases (13/24), both study areas were involved. The application of topical negative-pressure therapy in combination with a dermal substitute could potentially improve graft take and the rate of wound healing^29^ by reducing shearing force and limiting seroma.^11,30,31^ Simultaneously, this could reduce the risks of slow wound healing and bacterial colonization.

Three months postsurgery, a limited number of biopsies were analyzed using immunohistochemistry and RNA analysis.^32^ This analysis revealed that predominantly extracellular matrix pathways were activated in both treatment modalities. Further, neither remnants of the dermal matrix nor high concentrations of inflammatory cells could be identified by (immuno)histochemistry, indicating that complete remodeling had occurred at 3 months posttransplantation. Similar remodeling data were found in the earlier experimental studies that preceded this clinical evaluation.^16^

Scar elasticity parameters Uf and Ue as measured by the Cutometer were statistically better for dermal matrix-treated areas compared with standard treatment at 12 months. This outcome of dermal substitutes has also been observed in other studies.^33^ The results of the POSAS and DSM-II were similar for both treatments. When compared with scar parameters measured at 1 year posttreatment, data from the 6-year evaluation showed that scar maturation did not proceed much further beyond the 1-year time point for most parameters. Only scar erythema, measured by the DSM spectrometer, showed further improvement over time for both study areas. These data shed further light on the process of scar maturation: although scars may be almost fully matured after 1 year, scar color may require more time to reach full maturation.

The fact that relatively small differences were found in scar quality between the dermal matrix and standard of care-treated areas could be related to the fact that the researchers used relatively small expansion rates for the mesh grafts of ratio 1:1.5. Previous research indicates that especially in wounds treated with a dermal substitute combined with largely expanded grafts, the relative benefit of the dermal substitute becomes greater.^34^ Future studies should assess the ideal expansion required to optimize the benefits in the use of dermal substitutes for specific wound types (eg, small vs large burned areas). As part of such studies, researchers should also consider cost-effectiveness because there is a dearth of data on this subject in the field of skin-replacement materials. Potential benefits of dermal matrix application may include a reduced need for scar revision procedures and improved quality of life for the patient. Collection of such data, however, requires adequate registration, long-term follow-up, and a cost/benefit model for quality of life.

Currently, cell-seeded full-thickness skin substitutes are under development.^35^ Experimentally, the use of autologous cells in dermal and full-thickness skin substitutes yields better scar quality.^36,37^ Known limitations of these bioengineered products are their cost, long production time, and, most importantly, inability to be used off the shelf.^38^ Therefore, new developments in the field of off-the-shelf substitutes are warranted. Although the dermal matrix investigated in the present study showed some limitations in terms of the rate of wound healing, its safe use and objectified improved elasticity of scars encourage further investigations of substitutes for best uses, best structural characteristics, and concomitant therapies to further improve the outcome of wound healing and scar quality.

## CONCLUSIONS

The use of a novel dermal regeneration matrix in combination with an STSG in a one-step procedure for the treatment of full-thickness skin defects was found to be safe. Graft take was lower for dermal matrix plus STSG compared with STSG alone (similar to results observed for other dermal substitutes), and, subsequently, epithelialization took longer. However, several scar quality parameters, especially elasticity, tended to be better for the wounds treated with dermal matrix at 1 year postsurgery. However, the authors could not establish differences in scar quality after 6 years, indicating that scar maturation was probably reached earlier with the dermal substitute treatment.
